# A Corpus-Based Comparison of the Chief Executive Officer Statements in Annual Reports and Corporate Social Responsibility Reports

**DOI:** 10.3389/fpsyg.2022.851405

**Published:** 2022-04-22

**Authors:** Qingrong Liu, Bilal, Bushra Komal

**Affiliations:** ^1^Department of English, Wuhan University, Wuhan, China; ^2^School of Accounting, Hubei University of Economics, Wuhan, China; ^3^Business School, University of International Business and Economics, Beijing, China

**Keywords:** CSR reports, annual reports, CEO statements, semantic domains, corpus

## Abstract

This study presents a corpus-based comparison of the Chief Executive Officer (CEO) statements between annual reports and corporate social responsibility (CSR) reports. Using a corpus of 209 CEO statements from annual reports and CSR reports of Chinese companies, this study employs the Discourse-Historical Approach of critical discourse analysis to investigate the nomination strategies and key topics in these two related reports. The results showed that corporate leaders tend to have different priorities in annual reports and CSR reports. In annual reports, corporate leaders highlight the economic and pragmatic concerns of stakeholders to create a professionally capable and objective corporate image. In CSR reports, corporate leaders highlight the ethical concerns of stakeholders to create a socially responsible corporate image and adopt a more engaging and affiliative voice through the use of first-person pronouns. This study has significance in understanding the differences in the related genres of annual reports and CSR reports for the stakeholders.

## Introduction

Corporate communication plays a crucial role in establishing favorable relationships with stakeholders. In corporate communication, Chairman’s or the Chief Executive Officer (CEO) statement serves as an important means to project the image of the corporation and can reflect “the tone at the top” (Amernic et al., 2010). A plethora of studies have explored impression management in the CEO statements to project a positive corporate image (Conaway and Wardrope, 2010; Barkemeyer et al., 2014; Aerts and Yan, 2017; Boudt and Thewissen, 2019; Yan et al., 2019; Na et al., 2020). As a separate section placed at the beginning of annual reports and corporate social responsibility (CSR) reports, the CEO statements embody the major performance, plans, and missions of a company. Due to its great importance, the CEO statement has been an area of great interest in a wide range of fields, including accounting and applied linguistics.

Previous studies of the CEO statements are mainly taken from annual reports (Hyland, 1998; Bhatia, 2008; Amernic et al., 2010; Conaway and Wardrope, 2010; Patelli and Pedrini, 2014; Boudt and Thewissen, 2019; Yan et al., 2019). So far, only a few studies have investigated the CEO statements in CSR reports (Barkemeyer et al., 2014; Rajandran and Taib, 2014b; De-Miguel-Molina et al., 2019; Na et al., 2020). As the CEOs make statements in both the annual reports and CSR reports, it is of great value to explore how the CEOs adapt their discursive strategies and content in these two related genres. The motivation for this study came from our observation that some companies had the same or very similar CEO statements in annual reports and CSR reports. For instance, the CEO statements of Sinotrans Limited in 2018 were identical except for the introductory sentence.

In fact, annual reports and CSR reports can be seen as two related genres that address different stakeholders and convey different communicative purposes (Fuoli, 2018). In annual reports, the main stakeholders are investors and shareholders. By contrast, the stakeholders in CSR reports are more diverse and less focused. As different stakeholders have different expectations and interests, Pirson and Malhotra (2011) advised organization leaders to adjust information disclosure to the needs of different stakeholders, so as to build a trustworthy image effectively. To facilitate effective communication, the CEOs need to have genre knowledge and adjust their discourse to present an identity that best suits the needs of the audience and communicative purpose. Genre knowledge includes not only the right form of language, but also the awareness of choosing appropriate content “to a particular purpose in a particular situation” (Berkenkotter and Huckin, 1995). As the CEO statements are voluntary and unaudited, the CEOs have great freedom in the choice of content (Boudt and Thewissen, 2019). Therefore, the identification of recurrent themes in the CEO statements is important because it reflects the attention of corporate leaders (Amernic et al., 2010). This study aims to identify the interpersonal and ideational differences when CEOs address different stakeholders.

This study used a corpus of the CEO statements made by 87 Chinese companies covering a wide range of sectors to compare how companies adjust corporate messages in different contexts. We employed the Discourse-Historical Approach (DHA) developed by Reisigl and Wodak (2009) as the analytical framework, which integrates nomination strategies and key topics. This study also makes a methodological contribution. While previous studies in accounting have generally conducted thematic analysis manually through repeated close reading (Smith and Taffler, 2000; Amernic et al., 2010; Conaway and Wardrope, 2010; Marais, 2012), this study identifies recurrent themes in the CEO statements by using an automatic software Wmatrix (Rayson, 2008). There is now an increasing trend of using corpus linguistic methods in the analysis of corporate discourses (El-Haj et al., 2019). Compared with traditional manual coding through close reading, corpus-based approach is more efficient and can also avoid potential subjectivity in manual coding (Breeze, 2018). So far, computer-assisted analysis of accounting has been mainly conducted through the concordances of specific words, such as first-person pronouns (Lischinsky, 2011), modality markers (Aiezza, 2015), or through sentiment analysis (Barkemeyer et al., 2014; Boudt and Thewissen, 2019; Na et al., 2020). With a corpus of CEO statements from annual reports and CSR reports from Chinese listed companies, this study addresses the following research questions:

1. What are the nomination strategies in annual report and CSR report CEO statements?
2. What are the key topics in annual report and CSR report CEO statements?

The remainder of this article is organized as follows. After a general review of related literature, the methodology of the research will be presented followed by the presentation of results and discussion. The article ends with implications and recommendation for future research.

## Literature Review

The extant research has studied companies’ legitimization of CSR activities from the perspective of firm performance (Bin et al., 2020; Saha et al., 2020; Suler et al., 2021), stakeholder engagement for green products (Ionescu, 2021; May et al., 2021; Vãtãmãnescu et al., 2021), financial behavior of retail investors (Crişan-Mitra et al., 2020; Priem, 2021; Bin, 2022), and corporate communication in the recent years. Specifically, corporation communication has gained an immense attention in academia regarding how a company portrays its financial and sustainable performance and activities to various stakeholders (Bedford et al., 2022; Jiang and Park, 2022). As the snapshot of corporate communication in a year, the CEO statements in CSR reports have received less attention compared with their counterparts in annual reports.

### Chief Executive Officer Statements in Annual Reports

Previous studies have consistently revealed the significance of the CEO statements to users of corporate annual reports (Bournois and Point, 2006; Amernic et al., 2010; Mäkelä and Laine, 2011; Barkemeyer et al., 2014). Till now, many studies have investigated the influence of financial performance on the CEO statements in terms of linguistic structures (Clatworthy and Jones, 2006; Cen and Cai, 2013; Moreno et al., 2019) and readability (Bayerlein and Davidson, 2011; Wang et al., 2018).

Thematic analysis of the CEO statements is also an important area that has drawn immense attention. Prior studies have generally conducted content analysis to identify the prominent themes of the CEO statements. For instance, Bournois and Point (2006) analyzed 28 French CEO statements to explore the main themes (e.g., market, growth, and strategic plans, etc.) and provided recommendations to the CEOs for improving their statements to stakeholders. Amernic et al. (2010) conducted a close reading of the nine CEO statements from a British Petroleum company and identified some prominent themes, including the heroic theme to emphasize achievement and leadership, the theme of difficult business environment and future risk, business strategy, business philosophy, and the theme of trust. Jonäll and Rimmel (2010) synthesized three major themes, i.e., the company’s strength, strategies, and future plans in the CEO statements of three Swedish companies *via* the close reading method. Ngai and Singh (2014) analyzed the CEO messages of 234 companies from China and identified the themes of environmental factors, growth, operating philosophy, product/market mix, unfavorable financial reference, and favorable financial reference. In another study based on 32 CEO statements, Ngai and Singh (2018) identified company development, operating philosophy, company profile, business environment, corporate performance, and product and service as key themes.

While these studies explore the common themes of the CEO statements, some studies have considered the influence of different factors on the themes of the CEO statements. One of the factors considered is culture. For instance, Conaway and Wardrope (2010) compared annual report CEO statements between United States and Latin American companies and identified eight key themes, namely, financial reporting, expansion, external environment, customer relations, corporate governance, leadership, social responsibility, and mission or outlook. Despite similar themes, this study found that the Latin American CEOs displayed richer topics such as gratitude and regional political issues, which reflect their high-context culture. In addition to culture, another factor that is found to influence the theme of the CEO statements is financial performance. For instance, Smith and Taffler (2000) compared the themes in the CEO statements of low- and high-performance companies using a sample of British manufacturing firms. They noted that firms with poor performance disclosed more bad news such as losses, closures, and resignations, whereas firms with better performance portrayed more good news such as profits, dividends, and growth. Similarly, Clatworthy and Jones (2001) showed that profitable companies tended to discuss results and acquisitions, whereas unprofitable companies were inclined to discuss board changes. Bhana (2009) found that companies with improving performance highlighted good news, whereas companies with declining performance downplayed bad news. Geppert and Lawrence (2008) conducted a content analysis of the CEO statements in companies of high and low reputation and found that high repute companies concentrated more on realism using more concert words and present tense than companies with low reputation. These studies provide evidence to the view that corporate leaders are selective and strategic in their narrative reporting (Clatworthy and Jones, 2006).

### Chief Executive Officer Statements in Corporate Social Responsibility Reports

With growing concern for environmental and social responsibility, it has become a common practice for companies to make CSR reports (Aiezza, 2015; Conte et al., 2020; Lin, 2020). Studies have shown that CSR reports play an increasingly essential role in evaluating corporate reputation (Goodman et al., 2011; Hetze, 2016; Pérez-Cornejo et al., 2020). Despite the importance of CSR reports, the CEO statements in CSR reports are under-researched and deserve more attention.

Among the scant literature on the CEO statements in CSR reports, Grantham and Vieira (2018) conducted a case study of the CEO statements of ExxonMobil company in CSR reports from 2002 to 2013 and found that profit and planet themes varied over time due to external factors. Specifically, the CEOs’ focus on profit declined following the 2007 financial crisis and the theme of planet gained more attention following the 2010 BP oil spill.

Some studies have employed critical discourse analysis (CDA) to examine the rhetorical strategies that the CEOs use to portray corporations as agents of positive change to different stakeholders of the companies (Rajandran and Taib, 2014a,b). For example, Rajandran and Taib (2014b) used a sample of 27 CEO statements in CSR reports of Malaysian companies and identified six themes, i.e., achievement, identification, aspiration, disclosure, recognition, and appreciation. Using the same sample of Malaysian companies, Rajandran and Taib (2014a) investigated the language strategies of CEO statements in disclosing CSR performance. This study proposed three strategies in portraying corporations as compliant and responsible agents, namely, the categorization of participants in CSR events, types of evaluation, and temporal representation of CSR performance. Rajandran (2018) identified the main stakeholders in the Malaysian CEO statements from CSR reports and examined CEOs’ communicative strategies through language and image in interacting with these stakeholders.

### Comparison of the Chief Executive Officer Statements in Annual vs. Corporate Social Responsibility Reports

So far, prior literature suggests the influence of cultural and economic context on CEO statements’ language and content. In fact, other contextual factors such as audience and communicative purposes also deserve consideration.

Till now, only a few studies have compared the CEO statements between annual reports and CSR reports, the two related genres with different stakeholders in mind, especially from the perspective of content. Among the few pertinent studies, Marais (2012) compared the types of rhetoric in 90 French companies’ CEO discourses related to CSR performance, including the CEO statements from annual reports and CSR reports. This study shows that instrumental rhetoric is mainly used by the CEOs in annual reports, whereas values rhetoric is mainly employed in CSR reports. In other words, the CEOs employ different rhetoric strategies to communicate and legitimize their actions through the CEO statements. Mäkelä and Laine (2011) examined the content of two Finnish companies’ CEO statements in annual reports and sustainability reports. Their study showed that the CEO statements in the two genres reflected different types of discourse, i.e., economic growth discourse in annual reports and well-being discourse in sustainability reports. Despite its insightful contribution to the understanding of the CEO statements in annual reports and CSR reports, this study only investigated two Finnish companies through qualitative studies. A corpus-based analysis with a larger sample can test if the finding can be generalized and can also provide quantitative information concerning the distribution of specific topics. Barkemeyer et al. (2014) conducted a corpus-based study to compare the CEO statements’ sentiment in sustainability reports and annual reports from 34 companies in 10 years. The results indicate that the CEO statements in sustainability reports are more optimistic and certain than those in annual reports. This study highlights the value of using corpora in comparing the two related genres of annual report and CSR report CEO statements. This study will extend the corpus-based comparison between these two genres from sentiment analysis to thematic analysis.

## Methodology

### Data Sources

This study was based on a corpus of the CEO statements made by Chinese companies listed in the Hong Kong Stock Exchange^1^ concerning their performance in the annual year of 2018. The companies listed in the Hong Kong Stock Exchange were chosen because they were required to provide detailed disclosures of CSR reports since January 2016 under new listing rules (HKEX, 2015, 2019). The year 2018 was chosen because it was the latest time of conducting this study. In addition, 2018 marks the 40th anniversary of the reform and opening-up policy in China. It is also the first year to implement the guiding principles of the 19th National People’s Congress of the Communist Party of China, which officially launched a new era in sustainable development and environmental protection. The historical context highlights the integration of economic development and sustainable development.

As this study aimed at comparing the CEO statements in annual and CSR reports, the companies that issued both the annual reports and stand-alone CSR/environmental, social and governance (ESG) reports were selected. Reports with CSR and ESG titles were merged into the same CSR reports genre because of their similar communicative purpose of reporting social responsibility. The companies that integrated CSR reports in annual reports were excluded from analysis. Among the 261 listed companies, 171 companies issued stand-alone CSR reports concerning their performance in 2018. The English versions of the CSR reports and annual reports of these companies were downloaded from their disclosure documents^2^ in PDF format. The companies that did not issue the CEO statements in either annual reports or CSR reports were excluded from the data. Among the companies that presented stand-alone CSR reports, 87 companies issued the CEO statements in both the annual reports and CSR reports. In the cases that companies issued both the Chairman’s and President’s statements, both the statements were collected. Altogether, 209 CEO statements by 87 Chinese companies were compiled, with 109 CEO statements from annual reports and 100 from CSR reports.^3^ Appendix 1 shows the list of the selected companies and their industry distribution based on the China Stock Market and Accounting Research (CSMAR) database classification. It can be seen that the companies cover a wide range of sectors.

The CEO statements in annual and CSR reports were extracted from the downloaded PDF texts and pasted to word files for checking and cleaning. The words that were unidentified or made separate due to formatting were fixed by checking the original documents. Chinese characters that occurred in the files were deleted. After checking, the CEO statements were saved as TXT files.

Table 1 shows the general information of the CEO statements corpus. The statistics indicate that the CEO statements in annual reports tend to be longer than their counterparts in CSR reports.

**TABLE 1:** The general statistics of the Chief Executive Officer (CEO) statements corpus.

Genre types	Files	Size	Average word length
Annual reports	109	156,997	1440
CSR reports	100	79,601	796
Total	209	236,598	1132

### Data Analysis

For the analysis of the data, the DHA developed by Reisigl and Wodak (2009) was adopted as the analytical framework. The DHA belongs to the broad school of CDA. In CDA, discourse is regarded as a system of linguistic choices from which authors make decisions about inclusion and exclusion (Benwell and Stokoe, 2006). Language not only represents reality, but also constitutes social reality. The selection and highlighting of certain aspects of reality is referred to as “framing” (Entman, 1993). In discourse, framing is reflected by the inclusion of certain keywords, key topics, and phrases that are salient, which can be identified through corpus tools.

The DHA analytical framework consists of five main discursive strategies, namely, nomination, predication, argumentation, perspectivization, and intensification/mitigation. For this study, we focused on the discursive strategies of nomination and argumentation. Nomination refers to the naming of social actors. As annual reports and CSR reports present major achievements of the company to different stakeholders, we investigated how companies referred to themselves and what main stakeholders were referred to. According to stakeholder theory, the top management of a company is responsible to all the parties that have direct and indirect stakes in the company including investors, suppliers, creditors, customers, community, and environment, etc. (Freeman, 1984; Freeman et al., 2006). The CEOs put their utmost efforts to fulfill the needs to these stakeholders and provide them with information concerning their CSR activities, priorities, challenges, and achievements in a particular year through the CEO statements in annual and CSR reports (Barkemeyer et al., 2014; Ngai and Singh, 2014; Fehre and Weber, 2016; Rajandran, 2018). Argumentation strategies are realized through key topics and macrotopics consist of many specific subtopics.

Among the common corpus tools available, Murphy (2013) compared three types of representative software in the extraction of themes. Based on the comparative results together with close textual analysis of the concordances, Murphy (2013) argued that keyword analysis software (WordSmith) and key semantic domain analysis software (Wmatrix) displayed more robustness in “discerning dominant messages in a text” than sentiment analysis software (DICTION) (p. 77). Compared with keyword method, key semantic domain analysis such as Wmatrix (Rayson, 2008) is more synthetic and robust in that it has the advantage of grouping words that are similar in meaning into a single category. Therefore, we employed the corpus tool Wmatrix developed by Rayson (2008) to identify the CEO statements’ key topics in annual and CSR reports.

Wmatrix employed UCREL semantic analysis system (USAS), an automatic semantic tagging system, to assign a semantic domain tag to each word in a given text (see Rayson et al., 2004 for a detailed introduction to USAS). USAS is a multitier semantic tagging system that classifies words into 21 major semantic categories and 232 specific semantic fields (the full tag set and prototypical examples of each semantic field can be downloaded in the website: http://ucrel.lancs.ac.uk/usas/). Wmatrix allows users to either choose the reference corpus already provided by the software or upload their own reference corpus to perform analysis. In this study, the two corpora of the CEO statements were both uploaded to Wmatrix and chosen as each other’s reference corpus when making key semantic domain analysis. The total frequency of each semantic domain of a given corpus is calculated and compared with that in the reference corpus using log-likelihood (LL), which is a statistical test widely employed in corpus linguistics to measure if there is significant difference in item frequencies between two corpora (Rayson, 2003). The semantic domains that have a significantly higher frequency in the selected corpus than the compared reference corpus are key semantic domains, which are a good indicator of the prominent topics of the corpus under investigation. Those with LL value larger than 10.83 is considered to be significant at the level of 0.1% or *p* < 0.001.^4^

As corpus linguists suggest the investigation of word lists and key topics in combination with their concordances, so as to present a better picture of the context of the words and provide the nuances of a message (Rayson, 2008; Murphy, 2013), we used Wmatrix to generate the concordances and collocates of a search term. The analysis of the concordances and collocates of the keywords can help to identify their typical uses and patterns.

## Results and Discussion

This section compares the CEO statements in annual reports and CSR reports in nomination strategies and key topics and discusses their differences.

### Nomination Strategies in the Chief Executive Officer Statements From Annual Reports and Corporate Social Responsibility Reports

Nomination refers to the naming of social actors (Reisigl and Wodak, 2009). In this study, we focused on self-reference and main stakeholders.

The choice of self-reference forms serves as a powerful strategy for identity construction (Blitvich, 2010). They can project different identities of the in-groups and out-groups (Tajfel, 1981). In self-reference, the CEO statements in both the annual reports and CSR reports used the first-person plural pronoun and third-person self-reference. However, as seen from Table 2, there is a significantly higher frequency of third-person self-reference in the form of the *company* and the *group* in the annual reports, whereas there is a significantly higher frequency of the first-person plural pronouns *we* and *our* in CSR reports. When referring to the companies, the CEOs prefer the more detached form represented by *the company* or *the group* in the annual reports. The use of the third-person self-reference form is considered to be a more inanimate reference (Thomas, 1997) and delivers an institutional voice. It can project a more detached and objective “out-group” identity (Tajfel, 1981). By contrast, the CEOs tend to project a more inclusive and affiliative voice in CSR reports through the use of *we* and *our*. Compared with *the company* or *the group*, the plural form of the first person is more inclusive and engaging, which can “maximize the affective impact” by involving all the members of the organization (Lischinsky, 2011). Such use projects an “in-group” identity (Tajfel, 1981) and can build solidarity and affiliation with the audience (Aiezza, 2015). Some of the most frequent content words that collocate with *we* include *served*, *adhere(d)*, *continue(d)*, *actively*, *always*, *improved*, *supported*, *enhanced*, *promoted*, *committed*, and *developed*. Most of these collocates have a positive connotation, which project an optimistic, committed, and caring corporate image.

**TABLE 2:** Statistics of self-reference in annual report and corporate social responsibility (CSR) report CEO statements.

Item	Annual report		CSR report		LL	Sig.
	Freq.	Per 1000 words	Freq.	Per 1000 words		
We	1227	7.82	1247	15.67	−293.75	0.000
Our	876	5.58	574	7.21	−22.35	0.000
The group	717	4.57	155	1.95	109.69	0.000
The company	1285	8.19	357	4.49	112.27	0.000

The nomination difference between the CEO from annual and CSR reports is also reflected in the communication regarding stakeholders. To identify specific stakeholders, this study drew related research (Rajandran, 2018) and the concordances of some main stakeholders. The words referring to stakeholders were searched in the corpus in both their singular and plural forms. Table 3 presents the main stakeholders in annual and CSR report CEO statements.

**TABLE 3:** Statistics of words referring to stakeholders in annual report and CSR report CEO statements.

Item	Annual reports		CSR reports		LL	Sig.
	Freq.	Per 1000 words	Freq.	Per 1000 words		
Customer	422	2.69	228	2.86	0.59	0.441
Employee	119	0.76	309	3.88	–264.85	0.000
Shareholder	318	2.03	93	1.17	23.91	0.000
Environment	152	0.97	142	1.78	–26.82	0.000
Society	81	0.52	209	2.63	–178.25	0.000
People	82	0.52	162	2.04	–108.67	0.000
Director	134	0.85	40	0.50	9.44	0.002
Government	68	0.43	53	0.67	–5.37	0.020
Community	33	0.21	88	1.11	–0.21	0.000
Staff	72	0.46	40	0.50	–0.53	0.644
Investor	59	0.38	35	0.44	–3.86	0.465
Supplier	21	0.13	30	0.38	–0.04	0.839
Client	22	0.14	12	0.15	0.00	1.000

The four major stakeholders identified in Rajandran’s (2018) study, namely, the *community*, *customer*, *employee*, and *environment*, all had a high frequency in this corpus, despite some slight differences in the ranking. While community was found to rank first in Rajandran’s (2018) study of the Malaysia CEO statements, it had a lower rank in this corpus. One of the reasons may be attributed to the fact that community was one of the designated sections in CSR reports in Malaysia (Rayson et al., 2004), but it was sometimes subsumed in the broader category of society in Chinese CSR reports. In the CEO statements in Chinese companies, there was a high frequency of referring to society and the public, which express a similar meaning to community. Another reason may be due to the different size in sampling. In Rajandran’s (2018) study, only 32 CEO statements from 15 companies were included. With a much larger and wider sample of 209 CEO statements from 87 companies, this study identified a wider range of highly frequent stakeholders.

The comparison indicates that there is a significantly higher frequency of *shareholder* in the CEO statements from annual reports. This finding consistent with prior research suggests that the CEOs tend to highlight the main stakeholders in annual reports, especially the stakeholders with economic interests and decision power (Goodman et al., 2011). As Bartlett and Chandler (1997) rightly maintain, the general-purpose nature of annual reports makes it unlikely to “satisfy the widely differing information needs of a large body of shareholders.” In contrast, the CEO statements in CSR reports have a significantly higher frequency of *employee*, *environment*, *society*, *people*, and *community*. In CSR reports, the CEOs try to connect with a wider network of stakeholders, showing care and concern to internal employees and external stakeholders (Brennan et al., 2013; Ngai and Singh, 2014; Cooren, 2020). Thus, the comparative results suggest that the CEO statements in annual reports are more pragmatic oriented, whereas CSR reports are more value oriented and people oriented.

### Key Topics in the Chief Executive Officer Statements From Annual Reports and Corporate Social Responsibility Reports

The key topics were identified through the software Wmatrix, which can generate key semantic domains in a given corpus.

#### Key Topics in the Annual Report Chief Executive Officer Statements

**FIGURE 1:**
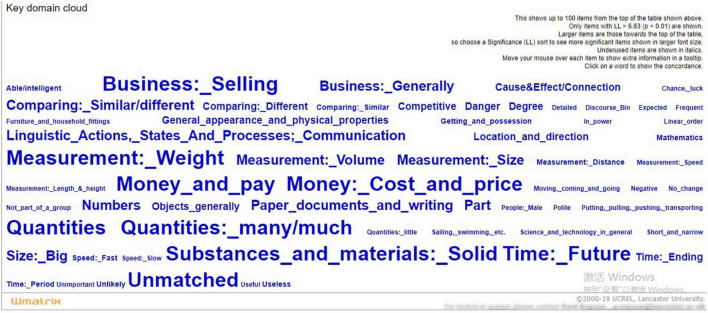
Key semantic domain cloud of the annual report Chief Executive Officer (CEO) statements.

Figure 1 shows the key semantic domain cloud of the CEO statements in annual reports. Following Rayson (2008) who listed the top 20 items when comparing the semantic domains of two related documents, we presented the top 20 key semantic domains in the descending order of LL value in Table 4.

**TABLE 4:** Top 20 key semantic domains in the annual report CEO statements.

Rank	USAS tag	Semantic domain	LL	Related words
1	I2.2	Business: selling	125.28	Market(s), customer(s), sales, marketing, retail, trade
2	N5	Quantities	113.91	Net, percentage, number
3	O1.1	Substances and materials: solid	109.1	Coal, copper, gold, zinc, fiber
4	I1.3	Money: cost and price	103.41	Cost(s), price(s), expenses
5	N5+	Quantities: many/much	97.72	Increase(d), increasing
6	I1.1	Money and pay	90.28	Shareholder(s), capital, assets, profit, investment, banking, credit, income, profitability, investors, dividend
7	Z99	Unmatched	89.69	Year-on-year, PRC
8	N3.5	Measurement: weight	83.65	Tonne(s)
9	T1.1.3	Time: future	75.45	Will, future
10	I2.1	Business: generally	72.92	Company, business(es), enterprises, economy
11	N5.1−	Part	49.18	Shares, segment(s)
12	Q1.1	Linguistic actions, states, and processes; communication	48.94	Representing
13	N3.4	Measurement: volume	46.09	Volume(s)
14	A6.1	Comparing: similar/different	45.82	Compared
15	Q1.2	Paper documents and writing	41.72	Record(ed), listed, listing, contracts, signed
16	N1	Numbers	41.33	2018, 2019, 2017, billion, million, trillion
17	N3.2+	Size: big	41.31	Growth, expansion, expand, big, expanded, large, grow(ing)
18	N3.2	Measurement: size	39.88	Capacity(es), scale, size
19	O2	Objects generally	32.88	Product(s), model(s), mechanism(s), container(s), equipment
20	M6	Location and direction	32.27	Overseas, internal

Semantic domains that share the same higher-level semantic category or have similar meanings are merged and discussed together. The top key semantic domains can be classified into the following main categories.

The first main semantic category includes *business: selling* (I 2.2), *business: generally* (I 2.1), *money: cost and price* (I 1.3), and *money and pay* (I 1.1). They share the same USAS tag I, which means money and commerce in industry (see http://ucrel.lancs.ac.uk/usas/usas_guide.pdf for the specific introduction to the USAS semantic tags). In the semantic domain *cost and price*, the representative word *cost* often occurs in phrases such as *cost reduction*, *cost-to-income ratio*, *low cost*, and *cost control*:

(1) The group earnestly promoted operation and management as well as **cost reduction** and efficiency improvement.

In the semantic domain *money and pay*, it is interesting to note that *shareholders* and *investors* are also included. The frequent occurrence of these two words suggests that the CEOs in annual reports are keenly aware of stakeholders that have financial relations with the companies.

The second key semantic category is about numbers and measurement represented by the semantic tag of N, including *quantities* (N5), *quantities: many/much* (N5+), *numbers* (N1), *measurement: weight* (N3.5), *measurement: volume* (N3.4), *measurement: size* (N3.2), *size: big* (N3.2+), and *part* (N5.1−). While *quantities*, *numbers*, and *measurement* show objective reports of numbers and measures, two other semantic domains in this main semantic category show positive evaluation, i.e., *quantities: many/much* (N5+) represented by *increase(d)*, *increasing*, and *size: big* (N3.2+) represented by *growth* and *expand(ed)*:

(2) In 2018, the bank achieved coordinated **growth** in quality and efficiency.

The frequent use of such words is consistent with previous studies’ finding that growth and expansion are a common theme in the annual report CEO statements (Bournois and Point, 2006; Amernic et al., 2010; Conaway and Wardrope, 2010; Mäkelä and Laine, 2011; Ngai and Singh, 2014).

The third main category includes *substances and materials: solid* (O 1.1) and *objects generally* (O2). The representative words in *substances and materials* are closely related to the specific industries of the companies. A study of the concordances of some representative words of *objects generally* shows that they typically occur in positive contexts, as can be seen in the following example:

(3) These products have boosted the **optimization** of the company’s **product structure** and the layout of strategic **new products** and **new special products**. The company has expedited the transformation and upgrading of high **value-added products** represented by canned beer and craft beer.

The fourth key semantic domain is *future*, represented by *will* and *future*. The most frequent right collocates of *will* is *continue* as shown in the following example:

(4) In the coming year, the bank **will** continue to follow the path of high-quality development.

The fifth key category includes *linguistic actions, states, and processes: communication* (Q 1.1) and *paper documents and writing* (Q 1.2), which share the semantic tag of Q, representing *language and communication*. In the semantic domain of *linguistic actions*, the most related word *representing* typically occurs in the context of *representing an increase of* and *representing a year-on-year increase of*:

(5) The healthcare service business’s revenue amounted to RMB 2555 million, **representing an increase of** 22.42% **compared** to 2017.

In the semantic domain of *paper documents and writing*, the representative word *recorded* often collocates with *revenue*, Renminbi (RMB), *million*, *billion*, and *growth*, which are also about financial performance.

Other semantic domains in the top 20 include *unmatched*, *comparison*, *location*, and *direction*. The *unmatched* domain includes words that cannot be classified into specific semantic domains. The most frequent word in the semantic domains of *comparison* was *compared*. It is interesting to note that *compared* often occurs in contexts to compare financial performance with the previous year as seen in example 5. The frequent use of such specific financial performance is consistent with previous finding that financial reporting is a common theme in the CEO statements from annual reports (Mäkelä and Laine, 2011). In the semantic domain of *location and direction*, the most frequent words are *overseas* and *internal*. Typical collocates of *overseas* include *market(s)*, *business(es)*, and *projects*. Typical phrases containing *internal* are *internal control*, *internal management*, and *compliance*. Such uses indicate that the CEOs are concerned not only about internal corporate governance, but also about international markets.

#### Key Topics in the Chief Executive Officer Statements From Corporate Social Responsibility Reports

**FIGURE 2:**
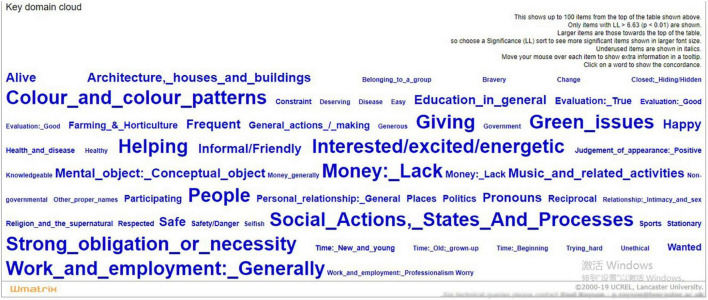
Key semantic domain cloud of the corporate social responsibility (CSR) report CEO statements.

Figure 2 shows the key semantic domain cloud of CSR report CEO statements. The specific statistics of the top 20 key semantic domains are shown in Table 5.

**TABLE 5:** Top 20 key semantic domains in the CSR report CEO statements.

Rank	USAS tag	Semantic domain	LL	Related words
1	W5	Green issues	317.59	Environment(al), ecological, energy conservation, pollution
2	I3.1	Work and employment: generally	254.76	Employee(s), work(ing), staff
3	I1.1−	Money: lack	251.3	Poverty, poor
4	S8+	Helping	247.83	Service(s), promote(d), protection, support(ed), promoting, serving, serve(d), cooperation, welfare, help(ed), benefit(s), supporting
5	O4.3	Color and color patterns	241.23	Green, blue
6	S6+	Strong obligation or necessity	161.36	Responsibility(ies), need(s), commitment, should
7	S1.1.1	Social actions, states, and processes	155.66	Social
8	S2	People	89.91	People
9	A9−	Giving	86.07	Provide(d), contribute(d), providing, contribution(s), donation(s)
10	X5.2+	Interested/excited/energetic	69.91	Active(ly), vigorously, proactively, energy, interests
11	Z8	Pronouns	64.08	We, our
12	N6+++	Frequent	54.12	Always
13	A15+	Safe	49.76	Safety, safe
14	P1	Education in general	45.12	Philosophy, education, training
15	K2	Music and related activities	40.59	Harmonious
16	S1.2.1+	Informal/friendly	40.43	Harmony, friendly
17	X4.1	Mental object: conceptual object	38.71	Concept(s), principle, dream(s), vision
18	E4.1+	Happy	38.25	Happiness, relief, happy
19	L1+	Alive	37.9	Life, lives
20	H1	Architecture, houses, and buildings	29.79	Build(ing), construction, built

These top key semantic domains in CSR report CEO statements can be classified into the following major categories.

The first main category concerns environment, including *green issues* as well as *color and color patterns* represented by *green*, which is also related to green issues, as can be seen in the following example:

(6) We made innovation in **green** insurance and financing services to facilitate **pollution** prevention.

The second main category is *work and employment*. The following example reflects the importance attached to employees in CSR report CEO statements:

(7) We are committed to creating a happy home for **employee** growth. We see **employees** as the key to achieving exceptional results and strive to create an enabling environment for **employees** by putting them first.

The third key category is related to *lack of money*, represented by *poverty* and *poor*. A detailed examination shows that the most common collocate of *poverty* is *alleviation*, which actually shows corporations’ concern for society (see example 8).

The fourth key category covers a wide range of semantic domains, including *helping* (S8+), *strong obligation or necessity* (S6+), *social actions*, *states*, and *processes* (S1.1.1), *people* (S2), and *informal/friendly* (S1.2.1+), which all share the semantic tag of S, meaning social actions, states, and processes. A typical example is provided below:

(8) In this year, we vigorously fulfilled our **social responsibility** by strengthening targeted poverty alleviation and actively engaging in rescue and relief work, instilling among the people a deep-rooted image of an enterprise with firm **commitment**.

The frequent use of such words is consistent with previous research finding that social responsibility is a common theme in the CEO statements from CSR reports (Marais, 2012). The frequent occurrence of the semantic domain of *people* also supports evidence from previous research that the CEO statements from CSR reports are people oriented (Grantham and Vieira, 2018).

Three other semantic domains are also related to corporations’ engagement with social responsibility. One is the semantic domain of *giving*, which shows corporates’ contribution to the society, as illustrated in the following example:

(9) The Bank **donated** more than RMB 300,000 to remote primary schools, villages, and towns, **donated** RMB 2 million to Zhengzhou Charity Federation, and **provided** financial assistance to poor university students for 3 consecutive years.

Another semantic domain related to social issues is *architecture*, *houses*, and *buildings*, represented by *build(ing)*, *construction*, and *built*. Typical phrases containing *construction* include *infrastructure construction* and *construction of ecological civilization* and typical words that follow *building* include *society* and *China*. This semantic domain is used metaphorically by comparing the construction of society to the building of a house:

(10) The year 2019 will be an important year in advancing the 13th Five-Year Plan and instrumental for securing a decisive victory in **building** a moderately prosperous society in all the respects.

The semantic domain of *alive*, represented by *life* and *lives*, is also related to social issues. A detailed study of the collocates and concordances shows that the most common phrase containing *life* is *a better life*, reflecting corporations’ concern for improving people’s living standards.

The fifth key category is *interested/excited/energetic*. This category is mainly about positive stance, represented by *active(ly)*, *vigorously*, and *proactively*. Two other keywords in this semantic domain are *energy* and *interests*. But, it should be noted that the common collocates of *energy* are *conservation*, *saving*, *reduction*, *emission*, *clean*, and *consumption*, which show concerns for environmental protection. As for the word *interests*, common phrases include *rights and interests* and *employees’ interests*. Thus, *energy* and *interests* in this category show corporations’ responsibility for the environment and employees.

The sixth category is *mental object*, especially conceptual object, represented by *concept(s)*, *principle*, *dream(s)*, and *vision*:

(11) We always adhere to the **concept** of green development.

Closely related to this semantic domain is *education in general*, represented by *philosophy*, *education*, and *training*. Typical phrases of *philosophy* include *new development philosophy*, *people-oriented philosophy*, and *philosophy of sustainable development*, similar to that of concept and principle in expressing the values and missions of the company. A study of the concordances of *education* shows that it mainly refers to the specific field that the company contributes to or the training of employees.

Several other semantic domains are related to the mental concepts of values and missions. One is the semantic domain of *safe*, represented by *safety* and *safe*. Common phrases include *safety production*, *safety management*, *safe production*, and *safe working environment*. Another semantic domain is *music and related activities*, represented by the word *harmonious*. A close examination shows that the word *harmonious* is used metaphorically to indicate agreeable relationship, as can be seen in the following example:

(12) We share the benefits with stakeholders and are dedicated to promoting healthy, sustainable, and **harmonious** development of the economy, society, and environment.

Another semantic domain related to mental objects and missions is the semantic domain of *happy*, represented by *happiness*, *relief*, and *happy*.

The semantic domain of *pronouns* represented by *we* and *our* is discussed in the nomination strategies in section “Key Topics in the Annual Report Chief Executive Officer Statements.” The identification of words with a wide range of word classes shows the robustness of Wmatrix in identifying different priorities in two corpora. The semantic domain of *frequent*, represented by the adverb *always*, concerns interpersonal meanings, which will be explained in detail in the following section.

#### Comparison of Key Topics in the Chief Executive Officer Statements From Annual Reports and Corporate Social Responsibility Reports

The comparison of the CEO statements between annual reports and CSR reports shows substantial differences in their key topics. The results corroborate Mäkelä and Laine’s (2011) findings that the CEO statements in annual reports tend to represent economic discourse of growth and profitability, whereas the CEO statements in CSR reports are more about social and well-being discourse. The differences also corroborate Fuoli’s (2018) argument that the CEOs in annual and CSR reports tend to portray different aspects of corporate identity.

In annual reports, there is a tendency to highlight business outcomes such as market expansion, sales revenue, market shares, and cost reduction. Such rhetoric based on rational arguments with a focus on economic benefits to stakeholders appeals to readers’ logos to achieve pragmatic legitimacy (Marais, 2012). Using specific numbers and measurement to present growth and improvement over the previous year can enhance investors’ confidence and portray a competent and pragmatic corporate image. As Clatworthy and Jones (2006) point out, successful companies are more likely to present their comparative results. It is interesting to note that despite the overall pattern of showing goods news through specific numbers and comparison, some companies also reported decreased profits or sales compared with the previous year. Although reports about financial loss or decline may influence shareholders or investors’ confidence about the company’s financial performance (Lin, 2020), factual reports can nevertheless project a credible and trustworthy corporate image (Lamond et al., 2010).

By contrast, the CEOs in CSR reports tend to highlight green issues, social responsibility, and people’s well-being. They demonstrate adherence and commitment to the concept of green development, people-oriented development, and sustainable development. Such rhetoric of values appeals to readers’ pathos to achieve moral legitimacy (Marais, 2012). By highlighting the issues that are based on moral values, the CEOs project an ethical, caring, and responsible corporate image (Ozdora-Aksak and Atakan-Duman, 2015; Nwagbara and Belal, 2019). In addition, the frequent occurrence of *harmonious* and *harmony* in CSR report CEO statements echoes corporate responses to the Chinese government’s initiation in 2006 of the idea of building a harmonious society (Marquis and Qian, 2014). The use of these intertextual links to government discourse reflects Chinese corporations’ effort to attain political legitimacy (Marquis et al., 2017; Tang et al., 2018). Aside from verbal statements, the CEOs also list specific social responsibility initiatives, including their concrete efforts in pollution prevention, strengthening employee training and development, creating a safe working environment, participating in poverty alleviation activities, and making donations. The presentation of concrete CSR initiatives can help corporations project a trustworthy corporate image.

In addition to the distinct differences at the level of ideational meaning, the comparison between the CEO statements from annual and CSR reports also reveals differences at the level of interpersonal meaning, particularly in the use of *always* and *will*. The word *always* occurs more frequently in CSR report CEO statements, but the word *will* occurs more frequently in the annual report CEO statements. The word *always* can be seen as a booster in the field of metadiscourse, which highlights the writer’s certainty (Hyland, 2005) and can reflect the company’s “constant commitment” (Aiezza, 2015). The more frequent use of this word in CSR report CEO statements is consistent with Barkemeyer et al.’s (2014) finding that the CEOs displayed more certainty in CSR reports than in annual reports. Compared with past facts, future-looking statements are considered to be less reliable (Aiezza, 2015). Quite a few studies suggest that less profitable companies tended to use more future references as impression management strategies (Clatworthy and Jones, 2006; Cen and Cai, 2013). Although this study did not consider the company’s profitability, their frequent occurrence indicates that future outlooks are still an important move in the CEO statements from annual reports. They can also convey the determination and good intention of the company. In addition, the common phrase *will continue to* implicitly expresses the company’s successful past and constant commitment.

## Conclusion

This study compared the nomination strategies and key topics of the CEO statements in annual reports and CSR reports made by Chinese companies using the DHA of CDA. The results show that corporate leaders try to project a positive corporate image in both the genres, but tend to have different priorities. In annual reports, the CEOs aim to highlight the economic and pragmatic concerns of stakeholders to create a professionally capable and objective corporate image. In CSR reports, the CEOs tend to highlight the ethical concerns of stakeholders to project a socially responsible corporate image and adopt a more engaging and affiliative voice through the use of first-person pronouns to construct a caring corporate image.

This study has significance in understanding the differences in the related genres of annual report and CSR report CEO statements and can shed light on projecting positive corporate images to different audiences. In annual reports, the CEOs portray shareholders’ wealth maximization objective by putting more stress on money, quantities, and growth discourse to the current and prospective investors of the company. In contrast, the CEOs in CSR reports emphasize green issues, people, service, and values to show their social care. The findings of this study have implications for top management in considering the aspects covered in the CEO statements delivered to different audiences as well as their weight of priorities. This study also has methodological implications. We admit that we have identified some similar basic themes such as business growth in annual reports and green issues in CSR reports to prior studies, which adopted manual coding (Mäkelä and Laine, 2011; Marais, 2012). This consistence in general findings highlights the advantages and robustness of corpus-based automatic analysis over manual coding. This approach can, thus, be extended to the analysis of other accounting discourse. The automatic semantic analysis provides new avenues for future research of accounting narratives, which can help to identify the key themes or key topics of given texts (Bostan et al., 2020). In this study, we have focused on two discursive strategies of the DHA, namely, the nomination strategies and the key topics. As a comprehensive and powerful analytical framework, the DHA has been mainly applied in political discourse (Reisigl and Wodak, 2009). Other discursive strategies of the DHA can be investigated in accounting discourse.

Through detailed analysis of the keywords in each semantic domain together with their concordances and collocates, we have identified some fine-grained topics, which can have implications to the CEOs as to what to include in their statements. In addition, we have also identified some unique themes and keywords, such as poverty alleviation and harmonious development with Chinese characteristics as compared to prior studies in Western context (Mäkelä and Laine, 2011; Bostan et al., 2020).

Admittedly, a few limitations in this study should be noted. First, despite the robust function of automatic semantic tagging, it should be pointed out that USAS has a precision rate of about 91% and there are still instances of inaccuracy mainly due to word sense disambiguation, which is a great challenge for semantic coding (Rayson et al., 2004). For some polysemous words, they may be assigned a semantic field tag based on frequency-based dictionaries and past tagging experience, which do not exactly match the meaning in the context. For instance, the word “building” is classified into the semantic category of “houses and architecture” based on its prototypical meaning. But, in example (10), it means “develop” in the specific context, which is used in its associative meaning derived from the prototypical meaning. Similarly, the word “harmony” is classified into the semantic category of “music and related activities” because harmony also has the meaning of “notes of music combined together in a pleasant way.” But, in example (12), it means “a state of peaceful existence.” That is to say, the automatic semantic tagging system may not recognize the metaphorical meaning or fine-grained meaning of certain words that are polysemous. This is also one of the reasons why this study investigates the context of keywords through concordance and collocates to better interpret their meanings in contexts. Second, our findings are limited to a cross-sectional analysis of the CEO statements in annual and CSR reports of Chinese companies in a single year. Future studies can be extended to multiple years and other countries as the CEOs’ preferences for communicating with stakeholders might vary with changes in the external environment. Finally, it should also be pointed out that this study is based on textual analysis of the CEOs’ communication with stakeholders in annual reports and CSR reports, without considering their effectiveness. Thus, we urge upcoming studies to conduct qualitative research by interviewing different stakeholders to explore their perceptions and opinions of the effective CEO statements.

## Data Availability Statement

The original contributions presented in the study are included in the article/supplementary material, further inquiries can be directed to the corresponding author/s.

## Author Contributions

QL and Bilal contributed to conception and design of the study. BK organized the database. QL performed the statistical analysis. QL, Bilal, and BK wrote the first draft of the manuscript. All authors contributed to the manuscript and approved the submitted version.

## Conflict of Interest

The authors declare that the research was conducted in the absence of any commercial or financial relationships that could be construed as a potential conflict of interest.

## Publisher’s Note

All claims expressed in this article are solely those of the authors and do not necessarily represent those of their affiliated organizations, or those of the publisher, the editors and the reviewers. Any product that may be evaluated in this article, or claim that may be made by its manufacturer, is not guaranteed or endorsed by the publisher.

## Footnotes

1 The listed companies can be found in the following website: https://www.hkex.com.hk/market-data/statistics/consolidated-reports/china-dimension?sc_lang=en.

2 The annual reports and CSR reports were downloaded in the following website by entering the stock code of each listed company: https://www1.hkexnews.hk/search/titlesearch.xhtml?lang=en.

3 Among the 87 companies, 22 companies issued both the Chairman’s statement and President’s statement in annual reports and 13 companies issued both the Chairman’s statement and President’s statement in CSR reports.

4
https://ucrel.lancs.ac.uk/llwizard.html

## Appendix 1

**APPENDIX TABLE 1:** List of the selected companies.

Industry	Company
Consumer goods (10)	Livzon Pharmaceutical Group Inc.
	YiChang HEC ChangJiang Pharmaceutical Co., Ltd.
	BAIC Motor Corporation Ltd.
	WuXi AppTec Co., Ltd.
	Weiqiao Textile Co. Ltd.
	Shanghai La Chapelle Fashion Co., Ltd.
	Dongfeng Motor Group Co., Ltd.
	Tsingtao Brewery Co., Ltd.
	BYD Co., Ltd.
	Shanghai Fosun Pharmaceutical (Group) Co., Ltd.
Consumer services (5)	Guangshen Railway Co., Ltd.
	Wenzhou Kangning Hospital Co., Ltd.
	China Eastern Airlines Corporation Ltd.
	Beijing Capital International Airport Co., Ltd.
	Air China Ltd.
Energy (6)	SINOPEC Engineering (Group) Co., Ltd.
	China Petroleum & Chemical Corporation
	PetroChina Co., Ltd.
	China Shenhua Energy Co., Ltd.
	Yanzhou Coal Mining Co. Ltd.
	China Coal Energy Co., Ltd.
Financials (30)	China Reinsurance (Group) Corporation
	Guangzhou Rural Commercial Bank Co., Ltd.
	Bank of Tianjin Co., Ltd.
	China Development Bank Financial Leasing Co., Ltd.
	Shandong International Trust Co., Ltd.
	Jiangxi Bank Co., Ltd.
	Shengjing Bank Co., Ltd.
	Bank of Gansu Co., Ltd.
	Chongqing Rural Commercial Bank Co., Ltd.
	Huishang Bank Corporation Ltd.
	Harbin Bank Co., Ltd.
	Bank of Jiujiang Co., Ltd.
	Bank of Zhengzhou Co., Ltd.
	GF Securities Co., Ltd.
	China Construction Bank Corporation
	China CITIC Bank Corporation Ltd.
	Agricultural Bank of China Ltd.
	New China Life Insurance Co., Ltd.
	The People’s Insurance Company (Group) of China
	China Cinda Asset Management Co., Ltd.
	China Zheshang Bank Co., Ltd.
	PICC Property and Casualty Co. Ltd.
	China Life Insurance Co., Ltd.
	Bank of Communications Co., Ltd.
	Bank of Qingdao Co., Ltd.
	China Merchants Bank Co., Ltd.
	Bank of China Ltd.
	China Everbright Bank Co., Ltd.
	Huatai Securities Co., Ltd.
	Postal Savings Bank of China Co., Ltd.
Industrials (15)	Beijing Jingcheng Machinery Electric Co. Ltd.
	Zhejiang Expressway Co., Ltd.
	Sinotrans Ltd.
	Dongjiang Environmental Co., Ltd.
	Datang Environment Industry Group Co., Ltd.
	COSCO SHIPPING Holdings Co., Ltd.
	Xinjiang Goldwind Science & Technology Co., Ltd.
	COSCO SHIPPING Development Co., Ltd.
	Xiamen International Port Co., Ltd.
	China Energy Engineering Corporation Ltd.
	Qingdao Port International Co., Ltd.
	CRRC Corporation Ltd.
	China International Marine Containers (Group) Co., Ltd.
	Zhuzhou CRRC Times Electric Co., Ltd.
	Weichai Power Co., Ltd.
Information technology (6)	ZTE Corporation
	Capinfo Co., Ltd.
	Chengdu PUTIAN Telecommunications Cable Co. Ltd.
	Chanjet Information Technology Co., Ltd.
	China Railway Signal & Communication Corporation Ltd.
	Yangtze Optical Fibre and Cable Joint Stock Limited Company
Materials (4)	Shandong Chenming Paper Holdings Ltd.
	Aluminum Corporation of China Ltd.
	Zijin Mining Group Co., Ltd.
	China Molybdenum Co., Ltd.
Properties and construction (8)	China Railway Group Ltd.
	Red Star Macalline Group Corporation Ltd.
	Beijing Urban Construction Design & Development Group Co., Ltd.
	Metallurgical Corporation of China Ltd.
	China Machinery Engineering Corporation
	BBMG Corporation
	China Vanke Co., Ltd.
	Baoye Group Co. Ltd.
Utilities (3)	Beijing Jingneng Clean Energy Co., Ltd.
	China Suntien Green Energy Corporation Ltd.
	Sichuan Energy Investment Development Co., Ltd.
